# Action observation: mirroring across our spontaneous movement tempo

**DOI:** 10.1038/srep10325

**Published:** 2015-05-19

**Authors:** Laura Avanzino, Giovanna Lagravinese, Ambra Bisio, Luisa Perasso, Piero Ruggeri, Marco Bove

**Affiliations:** 1 Department of Experimental Medicine, Section of Human Physiology and Centro Polifunzionale di Scienze Motorie, University of Genoa, 16132, Genoa, Italy; 2 Department of Neuroscience, Rehabilitation, Ophthalmology, Genetics, Maternal and Child Health, University of Genoa, 16132, Genoa, Italy

## Abstract

During action observation (AO), the activity of the “mirror system” is influenced by the viewer’s expertise in the observed action. A question that remains open is whether the temporal aspects of the subjective motor repertoire can influence the “mirror system” activation.

Healthy subjects show a common spontaneous movement tempo (SMT) ranging around 2 Hz. First, we investigated the excitability of the left motor cortex (M1) during the observation of videos displaying a hand performing a finger motor sequence at a rate similar to, lower or higher than the individual spontaneous one. The highest M1 excitability was observed during the AO of movements performed at the rate similar to the spontaneous one.

A second experiment aimed at (i) modifying the SMT through training consisting of a 10-minutes video observation of finger movements at 3 Hz; and (ii) evaluating the effects of this training on M1 excitability. AO training induced a shift of the SMT towards the 3 Hz frequency, as well as a change in M1 excitability during AO. Our results suggest that the temporal properties of a movement are recognized during AO and that the temporal motor resonance can be modulated by means of AO-based training.

When we observe the actions of others, we activate the same neural circuitry responsible for planning and executing our own actions^1^,^2^. Previous studies have shown that during action observation (AO) we use specialized motor representations to understand the observed actions^3^. The familiarity of the observed action is directly correlated with the degree of activation of the “mirror system”. Indeed, processing observed actions involves matching these actions not only to a predefined perceptual template, but to actions that already exist in the individual’s motor repertoire such as listening rehearsed music for a musician or watching a well-practiced dance move for an expert dancer^3^,^4^,^5^,^6^,^7^.

In this context, a question that remains open is whether the temporal aspects of the subjective motor repertoire can influence the “mirror system” in the process of movement recognition. Our motor repertoire includes over-learned movements, which are well characterized in terms of temporal organization. Indeed, healthy subjects have a common spontaneous movement tempo (SMT) during the execution of a number of voluntary movements. SMT has been a popular field of study of Gestalt psychologists in the first half of the 20th century. It can be simply determined from subjects freely tapping out a rhythm with their hand or fingers; on average, it measures about 2 Hz and is also closely correlated with the cadence of walking^8^,^9^,^10^,^11^. Recently it was shown that passive listening to preferred motor tempo, which is strongly correlated with spontaneous movement tempo^10^, modulates cortical excitability^12^. Following these findings, we hypothesized that if the action observation process selectively recognizes the temporal characteristics of finger movements, then cortical excitability would change as a function of movement rate, showing the maximal motor resonance for the rate which is the closest to the SMT.

In Experiment 1, in order to elucidate the influence of SMT upon action observation, we investigated the excitability of the left primary motor cortex (M1) by means of transcranial magnetic stimulation (TMS), during the observation of videos displaying repetitive finger opposition movements performed with the right hand, at a rate similar to, lower or higher than the spontaneous one (i.e., 2 Hz). In order to disentangle whether the effect on M1 excitability was a generic entrainment phenomenon due to a periodic visual stimulus or an action-specific effect, a control experiment based on the observation of a video showing a metronome beating at different rates (i.e., 1 Hz, 2 Hz and 3 Hz) was also performed.

It has also been shown that it is possible to learn or enhance the performance of a specific motor skill simply by observing motor actions^13^,^14^. Bove and coworkers^11^ demonstrated in healthy subjects that, after watching a video of repetitive finger movements paced at 3 Hz for 10 minutes, individuals’ SMT shifted from around 2 Hz to a rate close to that of the video. If the execution-observation matching system recognizes the temporal characteristics of a movement, then it would seem reasonable to hypothesize that AO training could modify the motor resonance properties at cortical level for the learned movement, as a consequence of SMT change. In Experiment 2, the M1 excitability while watching a video of finger opposition movements performed at the same rate as the SMT was measured before and after an AO training session; the training session consisted of watching a 10-minute long video displaying the same finger opposition movements paced at a higher rate than the SMT (i.e., 3 Hz; 3 Hz-AO training). Results were compared to those obtained from a control group who watched nature documentaries without human agents for10 minutes (LANDSCAPE training). This control setting, already adopted in previous studies^11^,^15^,^16^, has been revealed neutral in inducing modification in motor behaviour^11^,^15^ or in cortical excitability^16^.

## Results

### Experiment 1

The Experiment 1 aimed at evaluating if AO could selectively recognize the innate temporal characteristics of a certain movement. To this aim, at first subjects were asked to perform a finger motor sequence (opposition of thumb to index, medium, ring and little finger) with the right hand at their spontaneous movement tempo for 60 s. Mean movement rate was evaluated by means of a sensor-engineered glove^17^ and a commercial software to process the glove data (Glove Analyzer System, GAS, ETT S.p.A., Italy). Participants had a mean spontaneous movement rate of 1.88 ± 0.16 Hz.

**Figure 1:**
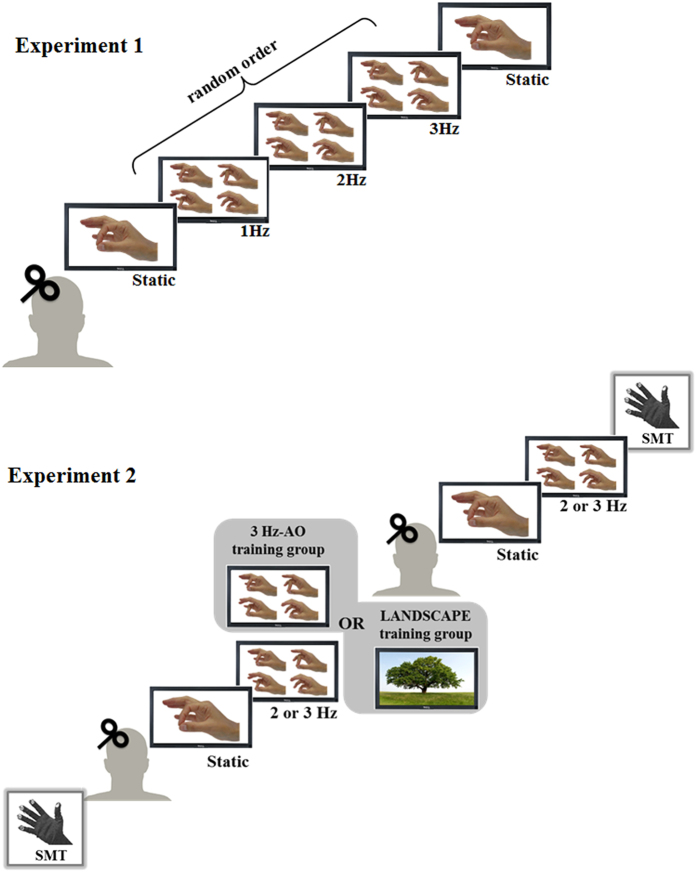
Experimental paradigm. Experiment 1. At the beginning of the experiment, participants’ spontaneous movement tempo (SMT) was evaluated by using a sensor-engineered glove. Then, the left M1 excitability was evaluated while subjects were watching videos showing either a right static hand or a right hand performing the finger motor sequence at 1 Hz, 2 Hz and 3 Hz. Experiment 2. SMT and left M1 excitability during AO were evaluated before and after an AO training (3 Hz-AO training or LANDSCAPE training). We acknowledge Ambra Bisio e Giovanna Lagravinese for drawing the images (hand and tree) presented in this figure.

Then, left M1 excitability was quantified using TMS. In particular, motor evoked potentials (MEPs) were recorded from the abductor pollicis brevis (APB) muscle while subjects were watching videos showing either a right static hand or a right hand performing the finger motor sequence at different rates: 1 Hz, 2 Hz and 3 Hz (the study design is depicted in Fig. 1). Cortical excitability during the observation of the video showing a right static hand was measured twice, at the beginning and at the end of the experiment in order to control for possible changes in left M1 excitability induced by the observation of the 1 Hz, 2 Hz and 3 Hz videos. The order of presentation of 1 Hz, 2 Hz and 3 Hz videos was randomized.

**Figure 2:**
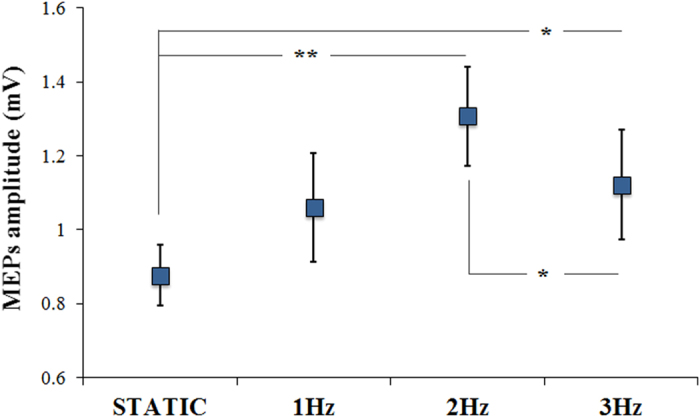
Left M1 excitability evaluated by the amplitude of MEPs during observation of a video showing either a static hand or a hand executing a finger motor sequence at different rates (1 Hz, 2 Hz and 3 Hz). 2 Hz was the rate more similar to the spontaneous one (i.e., 1.88 ± 0.16 Hz). Y-axis represents the MEPs amplitude in mV. Vertical bars indicate SE. * p < 0.05, ** p < 0.01.

The amplitude of MEPs collected during the static hand conditions at the beginning and at the end of the experiment did not differ between them and these MEPs values were averaged. The statistical analysis on MEPs data, based on a repeated measures analysis of variance (RM-ANOVA), showed a significant effect of Condition (static hand, 1 Hz video, 2 Hz video and 3 Hz video) (F (3,42) = 5.83; p = 0.002) suggesting that cortical excitability was differently modulated during the observation of videos showing finger opposition movements at different frequencies (Fig. 2). Post hoc analysis revealed that while subjects were observing the 2 Hz video (a rate of execution similar to the spontaneous one) or the 3 Hz video, MEPs amplitude were significantly larger than those measured during the observation of the static hand (static hand vs. 2 Hz video, p < 0.0001; static hand vs. 3 Hz video, p = 0.026). On the contrary, MEPs collected during the observation of the 1 Hz video were not significantly different from those collected during observation of the static hand (p = 0.142). Furthermore, M1 excitability during the observation of the 2 Hz video was significantly higher than during the observation of the 3 Hz video (p = 0.034).

### Control experiment

For the Control experiment, MEPs were recorded from the right APB muscle while subjects were watching videos showing either a static image of a metronome or a metronome beating at different rates: 1 Hz, 2 Hz and 3 Hz. The results of our Control experiment indicated that the effects observed in Experiment 1 were action-specific (Table 1). Indeed, RM-ANOVA showed that the effect of the main factor Condition was not significant (F (3,21) = 1.24; p = 0.32), suggesting that during the mere observation of a visual periodic stimulus (the metronome), the frequency of the metronome beat did not influence M1 excitability.

### Experiment 2

Experiment 2 was designed to modify the SMT by AO training with the aim to evaluate how this change could influence the motor resonance. As in the previous experiments, subjects wearing a sensor-engineered glove on their right hand were asked to perform the finger motor sequence for 60 s, at their SMT. As in the previous experiments, only subjects with a movement rate between 1.70 and 2.20 Hz continued the experiment. M1 excitability was tested applying TMS before and after the observational training. Subjects were divided in two groups who differed only for the observational training (AO training group and control group). Subjects in AO training group carefully watched a 10 minutes video of the finger motor sequence performed at a higher rate than the SMT, i.e. 3 Hz (3 Hz-AO training group). Subjects in the control group observed a 10 minutes video showing a nature documentary (LANDSCAPE training group). After the training, left M1 excitability was measured while subjects were observing (i) a static hand, (ii) the 2 Hz video, and (iii) the 3 Hz video (the same video used for the 3 Hz-AO training). The order of presentation of the 2 Hz and 3 Hz videos was randomized. Moreover, subjects were also asked to perform the finger motor sequence at their SMT for 60 s, in order to assess possible changes in the temporal features of the motor performance (Fig. 1).

**Figure 3:**
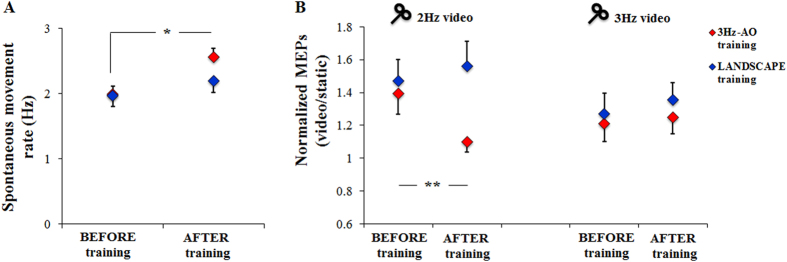
(**A**) Spontaneous movement rate and (**B**) MEPs changes induced by 3 Hz Action Observation (3 Hz-AO) training and LANDSCAPE training. Normalized MEP amplitude (MEPs recorded during observation of 2 Hz and 3 Hz videos with respect to MEPs recorded during the observation of static hand) is shown. Vertical bars indicate SE. Asterisks indicate that movement rate significantly increased after 3 Hz-AO training (**A**) and that normalized MEPs obtained during the observation of the 2 Hz video before 3 Hz-AO training were significantly higher than those evaluated during the observation of the 2 Hz video after 3Hz-AO training (* p < 0.05, ** p < 0.01).

Results from Experiment 2 are shown in Table 2 and Fig. 3. First, as in Experiment 1, RM-ANOVA on raw MEPs collected before training showed a significant effect of the main factor Condition (F (2,36) = 12.35, p < 0.001) and no effect of the factor Group or the interaction Group*Condition (p always >0.05) (Table 2). Post hoc analysis revealed that before training, in both groups of subjects, MEPs amplitude during the observation of the 2 Hz video or the 3 Hz video were significantly larger than those measured during the observation of the static hand (static hand vs. 2 Hz video, p = 0.001; static hand vs. 3 Hz video, p = 0.003) and that MEPs amplitude during the observation of the 2 Hz video was significantly higher than during the observation of the 3 Hz video (p = 0.045) (Table 2).

3 Hz-AO training induced a significant change in SMT and M1 excitability during AO. First, the movement rate significantly increased after 3 Hz-AO training, whereas it did not following LANDSCAPE training (Fig. 3A). The statistical analysis on glove data, based on RM-ANOVA, showed a significant interaction between Time (before and after 3Hz-AO training) and Group (3 Hz-AO training group and LANDSCAPE training group) (F (1,18) = 5.81, p = 0.027). Post hoc analysis revealed that movement rate significantly increased after 3 Hz-AO training (p < 0.001), whereas it did not significantly change after LANDSCAPE training (p = 0.75).

Regarding MEPs data, RM-ANOVA showed a significant interaction between Group, Condition (2 Hz video and 3 Hz video) and Time (F (1,18) = 4.45; p = 0.039) (Fig. 3B). Post hoc analysis revealed that normalized MEPs obtained during the observation of the 2 Hz video before 3 Hz-AO training were significantly higher than those evaluated during the observation of the 2 Hz video after 3 Hz-AO training (p = 0.006); conversely, normalized MEPs amplitude during the observation of the 2 Hz video was not influenced by LANDSCAPE training (p = 0.41). On the other hand, normalized MEPs amplitude during the observation of the 3 Hz video was neither modulated by 3 Hz-AO training (p = 0.67) nor by LANDSCAPE training (p = 0.66).

**Figure 4:**
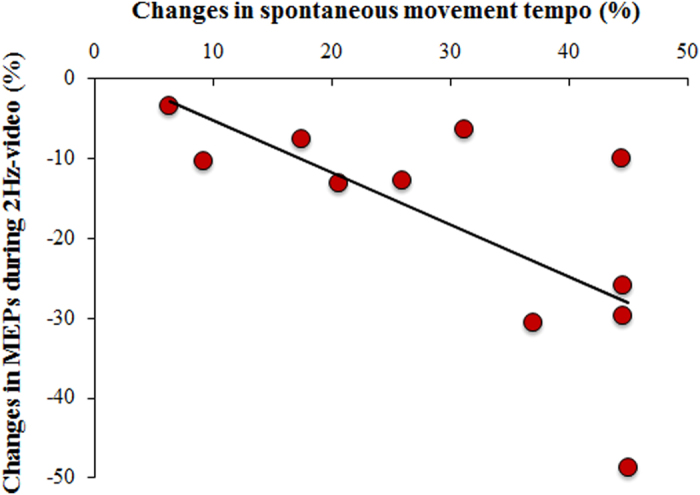
Correlation between changes in SMT and changes in MEPs amplitude induced by the 3 Hz-video training. Changes in SMT (X-axis) and in the left M1 excitability recorded during 2 Hz video observation (Y-axis) were evaluated as follows: SMT: (movement rate_post_ - movement rate_pre_)/movement rate_pre_*100; left M1 excitability = (MEPs amplitude_post_-MEPs amplitude_pre_)/ MEPs amplitude_pre_*100) (Spearman’s rho = -0.72; p = 0.013).

Finally, the correlation analysis between the changes in SMT induced by 3 Hz-AO training and changes in the left M1 excitability during 2 Hz video observation showed a significant negative correlation: the more the subjects increased their spontaneous movement rate after 3 Hz-AO training, the more the MEPs recorded during the observation of the 2Hz video were smaller with respect to baseline (Spearman’s rho = -0.72; p = 0.013) (Fig. 4).

## Discussion

In this work, we showed that during action observation the temporal properties of a specific movement are selectively recognized as belonging to the personal motor repertoire. We observed the larger motor evoked responses during the observation of a video showing finger movements performed at the rate that was similar to the individual spontaneous movement tempo.

Starting from the notion that the motor evoked potential facilitation is considered evidence of motor resonance effects^18^,^19^,^20^, here we demonstrated that motor resonance is influenced by observer’s expertise with the tempo of the observed rhythmic movements (temporal motor resonance).

The issue of “time” and motor resonance has been the subject of previous research in the field of action observation^21^,^22^. However, these studies aimed at unveiling whether the mirror neuron system recognizes the temporal dynamic of an observed movement as natural or not^21^, or at determining whether, during observation, motor pathways activation reproduces with high temporal fidelity the motor commands needed to execute the observed movement^22^. To our knowledge, whether the mirror neuron system is sensitive to the spontaneous tempo of rhythmic movements has been never studied so far. In accordance with the theoretical framework of embodiment, actions are mentally processed in a specific brain network that integrates observed actions of others with an individual’s personal motor repertoire allowing action understanding by motor simulation^4^,^23^. The same occurs for motor imagery, the mental process by which an individual rehearses or simulates a given action by recalling its motor plan. Indeed, subjective expertise in a specific movement influences the resonance of the motor system during motor imagery^23^ as well as during action observation^4^. We recently showed that, during mental simulations of sequential finger movements, the accuracy increases as the tapping rate gets closer to the individual’s SMT^24^. In line with all these findings, we propose that subjective motor expertise influencing motor resonance during action observation takes into account also the individual’s SMT. Here, we also showed that motor resonance is action-specific and not the consequence of a generic entrainment phenomenon. Indeed, observing a periodic stimulus (i.e., a metronome) moving at a rate similar to the SMT was not sufficient to modulate M1 excitability. Differently, evidence available in the literature suggests that passive listening to the preferred tempo (i.e. strictly correlated to SMT) modulates corticospinal excitability^12^. It has been shown that listening to rhythmic stimuli is associated with increases in BOLD activation of ventral premotor and supplementary motor areas^25^,^26^ and that increases in ventral premotor area activation are modulated by listening to preferred tempo^27^. Mental simulations of movement induced by the acoustic stimuli has been called into question to explain these findings^12^. The discrepancy between results on acoustic preferred tempo and the results of our control experiment using a visual periodic stimulus moving at the SMT, may be explained by different mechanisms involved in the two processes. Indeed, it has been shown that rhythmic movements are more strongly attracted to auditory rather than to visual stimuli^28^.

We are aware that our study design was limited to a sample group who had a SMT for rhythmic finger opposition movements centred on 2 Hz. Even if this choice was made in order to reduce the data variability, we think that future studies should address the influence of inter-individual variability in SMT on motor resonance in order to strengthen our hypothesis.

To better address the issue of SMT and motor resonance, we designed in this work a second experiment aimed at modifying the SMT by a training session based on action observation and at evaluating whether and how this training could affect the temporal motor resonance of M1. Firstly, we found that an AO training session based on the observation of finger opposition movements with temporal features (3 Hz movement rate) different from those produced in a spontaneous condition was able to induce a behavioural change in the SMT, in accordance with a previous work^11^. Furthermore, we showed that the changes in SMT induced by AO training significantly influenced the temporal motor resonance at cortical level. The motor evoked potentials recorded during the 2 Hz video presentation after the training were less facilitated than those recorded during the same experimental condition but before the training. Interestingly, correlating the changes in SMT with those induced in temporal motor resonance in left M1, we found that the greater the SMT change, the lower the M1 resonance with the 2 Hz video. This finding indicates that the degree of motor resonance of M1 with respect to SMT changed as a function of the shifting of SMT after the 3 Hz-AO training. In other words, the more the subjects learned “new” temporal features of movement, the less the M1 excitability increased during observation of movements executed at the individual baseline SMT. These results indicate that a new temporal structure of movement may be acquired by observation and that this learning process is accompanied by changes in cortical excitability. Notably, while the ability to resonate with the SMT (2 Hz video) changed after 3 Hz-AO training, no change in the cortical excitability associated to the 3 Hz video observation was observed after training. This result may suggest that a unique training session cannot be sufficient to change and consolidate a new temporal motor repertoire, also in term of motor resonance at a cortical level. However, whether temporal motor resonance can be “shifted” by multiple AO training sessions needs to be elucidated. It is worth noting that, for the AO training session, we decided to use a video showing finger movements performed at a higher rate than the spontaneous one (i.e., 3 Hz). Our findings suggest that AO training exerted an effect on SMT in terms of behaviour and motor resonance. Theoretically, similar results could have been achieved by training subjects with a video showing finger movements performed at a lower rate than the spontaneous one (i.e., 1 Hz) and this issue deserves to be addressed in future studies.

In the present study we did not plan specific experiments to directly test the neural structures implicated in the recognition of the innate temporal features of movement. Motor resonance effects, as reported in the current experiments, are typically proposed to occur from the activity of mirror neuron regions enhancing M1 excitability via excitatory cortico-cortical or subcortical- cortical connections^29^. The temporal processing of kinematics compatible with the human motor repertoire may rely on a common neural network that encodes the kinematics of both observed and executed movements^30^. For instance, it is well accepted that the superior temporal sulcus area (STS) is responsible for recognizing biological kinematics^31^. Therefore, we can assume that the temporal information could be sent from the STS to the fronto-parietal mirror neurons system^32^,^33^. Furthermore, basal ganglia and their inter-related cortical areas seem to be involved in SMT^34^. In the absence of external cues, SMT has to rely on internally generated, temporally regular pacing information. Such internal pacemaker function most likely engages the pre-supplementary motor area (pre-SMA) and its connections to basal ganglia. The pre-SMA contributes to the planning and initiation of simple and complex action sequences, and its recruitment is strongest when actions are freely chosen^35^. Starting from the notion that the basal ganglia network may be a part of the human “mirror neuron” system^15^,^36^, this network could be a good candidate in SMT recognition during action observation.

Here, for the first time we demonstrated that, during action observation, a brain network is responsible of selectively recognizing the temporal properties of a specific movement as belonging to the personal motor repertoire. This finding is in line with a recent work showing that TMS interference on motor simulation processes can impair the temporal coordination with other individuals^7^, highlighting the significance of the temporal properties of a trained movement during the processing of actions reproduced by other individuals.

Moreover, following AO training, subjects could implicitly learn the temporal characteristics of a movement, modifying their SMT and consequently their motor resonance behaviour. These results may give new hints for the design of new tailored protocols based on action observation in order to restore movement when it is lost or to change the pathologically modified temporal properties of a certain movement. We suggest that patients, who need to re-learn a specific movement should need to observe the same movement reproduced at their own SMT, in order to facilitate as much as possible the motor cortical activity through motor resonance, and trigger the motor memory formation process. On the other hand, when the movement repertoire is still present but the temporal properties are deteriorated (e.g., bradykinesia in Parkinson’s disease), the observation of a movement reproduced at a higher rate than the spontaneous one can be beneficial for these patients^15^. Also, in sports and music subjects have to learn new temporal properties of movements for specific body segments in order to correctly perform a skilled motor action. Available literature on action observation in musicians and elite sport players suggests that a particular experience with a sensorimotor task leads to action- perception coupling mechanisms that are stronger than in control subjects and that have a behavioural relevance in terms of superior ability in executing complex actions and making perceptual predictions^7^,^37^,^38^,^39^. In this case, action observation in combination with motor practice could theoretically boost and make the learning process faster with respect to motor practice alone.

## Methods

### Participants

A total of 40 subjects gave their consent to the participation to the study.

Five subjects were discarded since their SMT did not fit the inclusion criteria of this study. Given that the video displaying the finger motor sequence at 2 Hz was considered as the “SMT video”, only subjects with a SMT for finger opposition movements between 1.70 and 2.20 Hz were included in the study. Indeed, even if SMT measures on average about 2 Hz, inter-individual variability^6^ could exert a strong effect on our results; therefore, we decided to include in our TMS study only subjects whose SMT is very near to our SMT video (i.e., 2 Hz).

Fifteen healthy subjects (8 males, 7 females; mean age 21.9 ± 2.2) participated in Experiment 1, whereas 20 subjects (12 males, 8 females; mean age 24.1 ± 4.6) participated in Experiment 2; in particular in the Experiment 2, 11 subjects belonged to the 3Hz-AO training group and 9 subjects belonged to the LANDSCAPE training group. Eight additional healthy subjects with a SMT for finger opposition movements between 1.70 and 2.20 Hz (4 males, 4 females; mean age 24.9 ± 4.2) participated to the Control experiment.

Subjects had no history of psychiatric / neurological illness and no contraindication to TMS. The methods carried out in this work are in accordance with the approved guidelines. All participants were naïve to the purpose of the experiment and they gave written informed consent before participation. The experimental protocol was approved by the ethics committee of the University of Genoa and was carried out in agreement with legal requirements and international norms (Declaration of Helsinki, 1964). Right hand dominance was evaluated by the Edinburgh Handedness Inventory^40^.

### Experimental procedures

#### Motor task

Subjects seated in a comfortable chair in a quiet room and wore the sensor-engineered glove on their right hand. The motor task consisted in the motor execution of two repetitions of the same sequence of finger opposition movements (opposition of thumb to index, medium, ring, and little finger) lasting 60 s, separated by a 2-minutes rest. An eyes-closed paradigm was chosen to rule out the possibility of confounding effects attributable to visual information.

#### Electromyographic (EMG) recording

EMG activity was recorded from APB muscle, using silver disc surface electrodes. The ground electrode was placed at the elbow. The EMG signals were amplified, with a gain of 3000, filtered with a bandwidth ranging from 10 Hz to 1 kHz, analogue-to-digital converted at a sampling frequency of 2 kHz and fed into a personal computer by means of the MP100 acquisition system (BIOPAC Systems Inc., Santa Barbara, CA, USA). Each recording epoch lasted 400 ms, of which 100 ms preceded the TMS. Trials with background EMG activity were excluded from analysis (approximately 5%).

#### Transcranial magnetic stimulation

TMS was performed with a single Magstim 200 magnetic stimulator (Magstim Company) connected with a figure-of-eight coil (wing diameter: 70 mm). The coil was placed tangentially to the scalp with the handle pointing backwards and laterally at 45° to the sagittal plane inducing a postero-anterior current in the brain. We determined the optimal position for activation of the right APB muscles by moving the coil in 0.5 cm steps around the presumed motor hand area. At the beginning of each experiment, the stimulus intensity needed to evoke a MEP of approximately 0.8–1 mV peak-to-peak amplitude was defined (S1 mV). In every condition we collected 25 MEPs from right ABP at S1 mV.

#### Videos

For Experiment 1, different video clips were presented on a 19-inch screen located 60 cm from the subjects. Video clips showed either a right static hand or a right hand executing a finger motor sequence (opposition of thumb to index, medium, ring and little finger) at different rates. Each movie clip was obtained by filming on a white background the right hand of a human demonstrator from a third-person perspective who performed the finger motor sequence paced with a metronome at 1 Hz, 2 Hz and 3 Hz. The sound of the metronome was not listened by the participants. Each recorded video was continuously repeated until 25 MEPs were collected. To exclude possible confounding effects on evoked responses due to the amount of the observed finger aperture^41^, TMS stimuli were always administered at the end of the closing phase. Particularly, a custom-made MatLab software managed the synchronization between the presentation of the visual stimulus and the delivering of the magnetic stimulation. The magnetic stimulus was delivered when movement observation corresponded to the fifth finger-tapping movement while the hand was closing. Noteworthy, the inter-stimulus interval was always larger than 4 s to avoid any adaptation process to TMS. For the Control experiment, a video was used showing a metronome beating at 1 Hz, 2 Hz and 3 Hz. No audio accompanied the video presentation. The video was continuously repeated until 25 MEPs were collected. For Experiment 2, the same videos of Experiment 1 were used. During AO training the 3 Hz video was continuously repeated for 10 minutes. During LANDSCAPE training, subjects watched nature documentaries without human agents for10 minutes (LANDSCAPE training). During video observation, subjects were instructed to attend the stimuli and no further particular instruction was given to the subjects during the training.

### Data analysis

Regarding glove data, for each trial we computed the duration (in milliseconds) of both the transition phase from one finger to the successive one of the sequence (Inter Tapping Interval, ITI) and the finger touching phase (Touch Duration, TD). Then, the movement rate was calculated as [1/(ITI + TD)]*1000 and expressed in Hertz. The mean value obtained from the two repeated trials in each session was used for the statistical analysis.

Measurements of MEPs were made on single trials. The amplitude of contralateral MEPs (right ABP muscle) was evaluated by taking the peak-to-peak difference in the raw EMG signals. Mean values of MEPs amplitude were calculated for each subject in each experimental condition.

For Experiment 2, MEPs data collected during observation of the 2 Hz and 3 Hz videos were normalized with respect to MEPs data collected during observation of the static hand image at each testing time (before and after AO training). To investigate a possible relationship between changes in motor resonance and changes in SMT induced by 3 Hz-AO training, a delta value, expressed in percentage, for both the SMT and the left M1 excitability recorded during 2 Hz video observation was evaluated as follows: changes in SMT: (movement rate_post_ - movement rate_pre_)/movement rate_pre_*100; changes in left M1 excitability: (MEPs amplitude_post_- MEPs amplitude_pre_)/MEPs amplitude_pre_*100.

### Statistical Analysis

For Experiment 1, a paired t-test was performed to compare MEPs amplitude collected during the static hand condition at the beginning of the experiment versus MEPs amplitude collected during the static hand condition at the end of the experiment (see Fig. 1). For Experiment 1 and for the Control experiment MEPs data were subjected to a RM-ANOVA with the factor Condition (static, 1 Hz video, 2 Hz video and 3 Hz video) as main within-subjects factor. For Experiment 2, to confirm data of Experiment 1 raw MEPs data collected before training were entered in a RM-ANOVA with the factor Condition (static, 2 Hz video and 3 Hz video) as within subjects factor and the factor Group (3 Hz-AO training group and LANDSCAPE training group) as a between–subject factor. Then, to assess the effect of training, normalized MEPs data were entered in a RM-ANOVA with Group (3 Hz-AO training group and LANDSCAPE training group) as a between–subject factor and Condition (2 Hz video and 3 Hz video) and Time (before and after 3 Hz-AO training) as within-subject factors. Regarding motor performance, RM-ANOVA with the factor Group (3 Hz-AO training group and LANDSCAPE training group) as a between–subject factor and Time as within subject factor (before and after 3 Hz-AO training) was done in order to assess possible changes in movement rate induced by AO training with the 10-minutes 3 Hz video. Post hoc analysis of significant interactions was performed by means of t-tests applying the Bonferroni correction for multiple comparisons when necessary. P-values of 0.05 were considered as threshold for statistical significance. Finally, the Spearman’s correlation coefficient was applied to assess any correlation between changes in spontaneous movement tempo and changes in MEPs amplitude during 2 Hz video observation induced by the 3 Hz-AO training. Statistical analysis was performed with SPSS 13.0. Values are expressed as mean ± S.E.

## Author Contributions

L.A. and M.B. conceived and designed the experiments. L.A., G.L. and L.P. performed the experiments. L.A., G.L., A.B. and M.B. analyzed the data. L.A., G.L. and M.B. interpreted the data and wrote the paper. A.B. and P.R. drafted the article or revised it critically for important intellectual content.

## Additional Information

**How to cite this article**: Avanzino, L. *et al.* Action observation: mirroring across our spontaneous movement tempo. *Sci. Rep.* **5**, 10325; doi: 10.1038/srep10325 (2015).

## Figures and Tables

**Table 1:** Data from Control experiment.

	**Static metronome**	**1 Hz video**	**2 Hz video**	**3 Hz video**
MEPs amplitude (mV)	1.15 ± 0.05	1.05 ± 0.11	1.16 ± 0.09	1.01 ± 0.08

Motor evoked potentials (MEPs) amplitude values (mean ± SE) recorded during the observation of videos showing either a static image of a metronome or a metronome beating at different rates: 1 Hz, 2 Hz and 3 Hz.

**Table 2:** Data from Experiment 2.

**CONDITION**	**GROUP**	
	**3Hz-AO training**	**LANDSCAPE training**
Static hand	0.94 ± 0.06	1.02 ± 0.12
2Hz video	1.35 ± 0.15	1.58 ± 0.25
3Hz video	1.21 ± 0.12	1.18 ± 0.25

MEPs amplitude (mean ± SE) recorded before training during the observation of a static hand video and during the observation of videos displaying a hand performing a finger motor sequence at a rate similar to the spontaneous movement tempo (SMT) or at 3 Hz. Here data from either the 3 Hz-AO training group and the LANDSCAPE training group are reported.
